# Asian American Women’s Experiences of Discrimination and Health Behaviors during the COVID-19 Pandemic

**DOI:** 10.1007/s10903-023-01558-2

**Published:** 2023-10-26

**Authors:** Katarina Wang, Alice Guan, Janice Seto, Debora L. Oh, Kathie Lau, Christine Duffy, Esperanza Castillo, Valerie McGuire, Michelle Wadhwa, Clifford G. Tepper, Heather A. Wakelee, Mindy C. DeRouen, Salma Shariff-Marco, Iona Cheng, Scarlett Lin Gomez

**Affiliations:** 1 Department of Epidemiology and Biostatistics, University of California, San Francisco, USA; 2 Asian American Research Center on Health, University of California, San Francisco, USA; 3 Department of Urology, University of California, Davis, Sacramento USA; 4 Division of Oncology, Stanford University School of Medicine, Stanford, California USA

**Keywords:** Asian American, COVID-19, Anti-Asian Racism, Discrimination, Health Behaviors

## Abstract

The COVID-19 pandemic exacerbated racism experienced by Asian Americans, especially women and older individuals. Little is known about how discriminatory experiences during the pandemic have influenced health behaviors among Asian Americans. Between 10/2021 and 6/2022, we surveyed 193 Asian American women in the San Francisco area. Participants were asked to report types of discrimination they experienced since March 2020. We explored bivariable associations of discrimination and changes in health behaviors and healthcare utilization. Most women were Chinese American (75%) and over 45-years-old (87%). The top three discriminatory experiences reported were being treated with less respect (60%), being treated unfairly at restaurants/stores (49%), and people acting as if they are better (47%). Chinese American women (vs. non-Chinese Asian American women) reported higher frequencies of being threatened/harassed (40% vs. 22%). Women who reported any discriminatory experience (vs. none) were more likely to report less physical exercise (42.7% vs. 26.3%) and canceling/rescheduling medical appointments (65.0% vs. 45.1%). Our findings begin to elucidate Asian American women’s experiences of discrimination since the pandemic and provide evidence of the harmful impacts of anti-Asian racism on health behaviors.

**Supplementary Information:**

The online version contains supplementary material available at 10.1007/s10903-023-01558-2.

## Introduction

Anti-Asian racism has a long-engrained history in the United States, and anti-Asian sentiments in this country have increased since the onset of the COVID-19 pandemic due to racist rhetoric, with 10,905 recorded anti-Asian hate incidents occurring between March 2020-December 2021 [1]. In July 2020, about 39% of Asian Americans reported experiencing more racial slurs following the pandemic [2]. Women and elders (aged 60+) have been disproportionate targets of anti-Asian hate incidents [3]. For instance, between March 2020-March 2021, Asian American women reported 2.2x more hate incidents than men [4]. In San Francisco specifically, anti-Asian hate crimes increased more than any other category of hate crime in 2021 compared to the prior year [5]. Though these reports relied on voluntary reports of hate incidents, they suggest that COVID-related discrimination is a critical and underexplored dimension of life for Asian American populations.

Discrimination has harmful consequences for health, and has been found to be negatively associated with chronic conditions (e.g., cardiovascular disease, cancer) and healthcare-seeking behaviors of Asian Americans [6, 7], especially elders [8]. Given the disproportionate burden of increased discrimination following COVID-19 among Asian American women and elders, there is a need to elucidate the impacts of COVID-related racism on health behaviors and healthcare utilization in this population. Thus, our present study describes Asian American women’s discrimination experiences during the pandemic and associations with health behaviors and healthcare utilization.

## Methods

Asian American women participating in a never smoker lung cancer case-control study in the San Francisco Bay Area between October 2021 and June 2022 were invited to participate in the COVID survey [276 of 305 (90%) completed the COVID survey]. Lung cancer cases were recruited from the Greater Bay Area Cancer Registry, physician referrals, and social media. Controls were frequency-matched to cases by age and ethnicity. The participants were female, Asian American, 21–90 years of age, and spoke either English, Chinese, Vietnamese, or Tagalog. This study was approved by the Institutional Review Board (IRB#19-29151) at University of California San Francisco, Zuckerberg San Francisco General, and California Committee for the Protection of Human Subjects. Informed consent was obtained from all study participants.

We measured discriminatory experiences following March 2020 using an adaption of the 8-item Everyday Discrimination Scale (EDS) from the California Health Interview Survey Discrimination Module, which has been found to be valid and reliable for assessing discrimination in diverse populations [9]. This adaptation to the EDS appended the phrase, “because you are Asian, Asian American or Pacific Islander” to all questions (e.g., ”In the past 6 months, how often have people criticized your accent or the way you speak because you are Asian, Asian American, or Pacific Islander?”) [9, 10]. We additionally asked women to select changes to health behaviors because of COVID-19 (e.g. getting more/less physical exercise, drinking more/less alcohol). Finally, we measured changes to medical appointments as affirmative responses to the question, “Since March 2020, have you had to reschedule or cancel a health care appointment due to the COVID-19 pandemic?” and concerns about attending medical appointments as affirmative responses to the question, “Have you worried about attending medical appointments because of fear of being threatened or harassed because of your race or ethnicity?”

We described the frequency of these experiences by age (> 60-years-old/≤60-years-old), and by Chinese ethnicity (Chinese/non-Chinese Asian American). We explored bivariable associations between discriminatory experiences and changes in health behaviors and healthcare utilization. Statistical analysis was conducted using Stata 16, and significance (p < 0.05) was evaluated using chi-square tests.

## Results

Most of the women (n = 193) were over 45 years old at diagnosis (87.1%) and Chinese American (73.1%). Other Asian American ethnicities (comprising 26.9% of the sample) included Filipina (10.9%), Asian Indian (3.6%), Japanese (3.6%), Vietnamese (2.6%), Burmese (n < 5), Korean (n < 5), Multiethnic (n < 5), and Thai (n < 5). Our sample did not have any Native Hawaiian or Pacific Islander individuals. Nearly three quarters (70.9%) of women reported at least one experience of discrimination because of their race or ethnicity.

### Distribution of Discriminatory Experiences

**Fig. 1:**
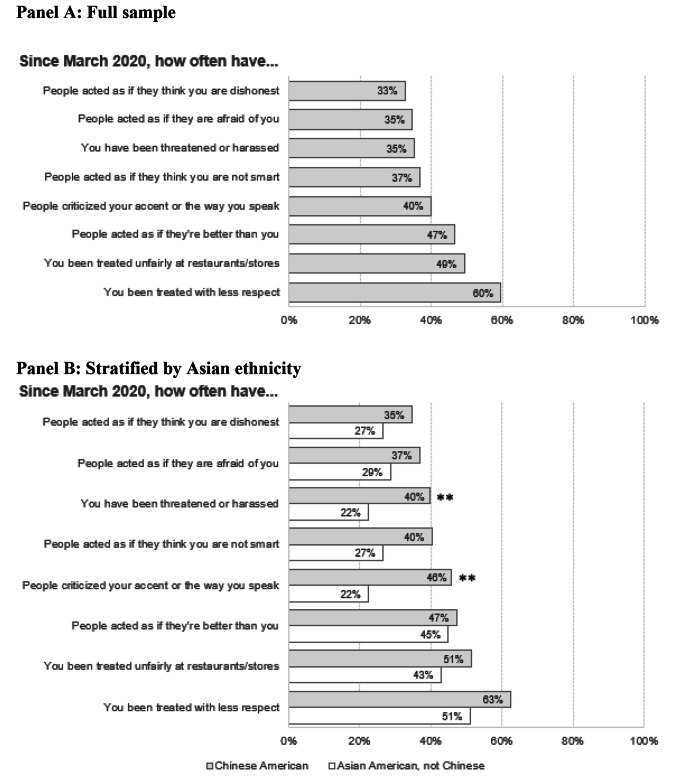
Frequency of specific discriminatory experiences among Asian American women overall, stratified by Asian ethnicity (n = 193). **p < 0.05; statistical significance was evaluated using chi-square tests

Across all types of discriminatory experiences, the most commonly reported were being treated with less respect (60%), being treated unfairly at restaurants/stores (49%), and people acting as if they are better (47%). We did not observe statistically significant differences in the distribution of discriminatory experiences by age at lung cancer diagnosis. However, compared to Asian Americans of other ethnicities, Chinese American women reported higher frequencies of being threatened/harassed (40% vs. 22%) and criticized for their accent (46% vs. 22%). (Fig. 1)

### Discriminatory Experiences, Health Behaviors, and Healthcare Utilization

Less than half of the sample reported worsened health behaviors including less physical exercise (37.8%), spending less time outdoors (39.9%), drinking more alcohol (5.2%). In contrast, more than half of the sample reported cancelling/rescheduling a medical appointment (59.2%). Women who reported any discriminatory experience (vs. none) were more likely to report less physical exercise (42.7% vs. 26.3%, p < 0.05) and were more likely to report canceling/rescheduling medical appointments (65.0% vs. 45.1%, p < 0.05). There were no bivariable associations with other health behaviors and healthcare utilization measures. (Table 1)

**Table 1:** Distribution of health behaviors and healthcare accessibility based on the number of self-reported discriminatory experiences since the pandemic

		Number of discrimination experiences		
	Overall N = 193	None N = 57	Any (1–8) N = 136	p-value
**Changes to health behaviors**				
Less physical exercise	37.8%	26.3%	42.7%	**
Less time spent outdoors	39.9%	33.3%	42.7%	
More alcohol	5.2%	4.5%	6.2%	
Other changes since pandemic^1^	18.7%	22.8%	16.9%	
**Changes to healthcare utilization**				
Cancelled/rescheduled md appt^2^	59.2%	45.1%	65.0%	**
Worried about attending medical appointment because of fears of being threatened or harassed	8.9%	8.8%	8.9%	

** p < 0.05; statistical significance was evaluated using chi-square tests

^1^ Other changes reported by participants included both negative (afraid to go to work, becoming more isolated, less interest in social interaction) and positive (better touch with friends over phone, enjoying more time with house pets) items.

^2^ 19 participants did not have scheduled appointments at the time of the survey.

## Discussion

Following March 2020, a startling majority (70.9%) of Asian American women in this study reported one or more discriminatory experiences, which is consistent with national studies that documented increased experiences of racism among Asian Americans [10]. Our results also suggest that Chinese American women reported higher frequencies of being harassed or criticized for their accent than Asian American women of other ethnicities. Although Asian Americans of all ethnicities experienced hate incidents following the pandemic [1], our findings are unsurprising given the increase in the use of anti-Chinese specific language (e.g., “Wuhan virus”, “Chinese virus”) on Twitter following the pandemic [11].

Women who experienced discrimination were less physically active. While different measures were used to evaluate discriminatory experiences, our finding aligns with the only other study conducted thus far that has explored the relationship between COVID-19-related racial discrimination and physical activity in the United States [12]. While national reports have suggested that Asian Americans most commonly experienced discrimination outside, in public spaces [1], we did not find differences in time spent outdoors on the basis of discriminatory experiences. Therefore, avoiding outdoor activity likely does not explain the associations we found. However, past research has linked COVID-19 related Anti-Asian racism to heightened anxiety levels [7], and individuals with anxiety tend to be more sedentary and engage in less physical activity [13]. To better understand whether this association is also seen among Asian Americans, further investigations are warranted. Future studies can delve into the potential relationship between discriminatory experiences, anxiety levels, and physical activity patterns among the Asian American population.

Furthermore, although the pandemic impacted healthcare utilization across the United States, our study revealed potential differences in healthcare seeking behaviors based on discriminatory experiences. Specifically, we found that women who experienced discrimination were more likely to cancel or reschedule medical appointments. However, women who experienced discrimination were not more fearful about attending medical appointments due to concerns of being threatened or harassed. This finding suggests that experiencing discrimination during COVID-19 as assessed in the EDS, such as being treated unfairly or criticized for one’s accent, was more damaging to our sample of Asian American women’s healthcare seeking behaviors than the fear of threats or attacks. To note, a considerable proportion of our study sample consisted of individuals with lung cancer, necessitating regular access to medical care. Although we did not find differences in the association between discriminatory experiences and healthcare appointment cancellations among those with and without lung cancer, missed healthcare encounters could contribute to decreased health screenings and exacerbate the burden of chronic diseases among Asian Americans.

Our work may have some limitations. The findings from our study may not generalize to all Asian Americans across the United States given the heterogeneity in this population. Since this is a descriptive and exploratory study, we did not adjust for potential confounders that could explain the differences we observed. Because the phrasing of our question related to medical appointments, it’s possible that it was physicians, not patients, who cancelled/rescheduled appointments.

We propose two key recommendations based on our study findings. Firstly, our research underscores the significance of data disaggregation and the necessity for further investigation into specific experiences of discrimination across different Asian American ethnic groups. While we observed variations in discriminatory experiences between Chinese and non-Chinese Asian Americans, the non-Chinese category in this study was heterogeneous, highlighting the need to investigate these constructs in studies with more participants from specific Asian ethnic groups. Secondly, the disparities in racism experiences highlight the importance of fostering solidarity among Asian American ethnic groups and other communities facing racial oppression. We advocate for the adoption of the “Four levels of solidarity” framework proposed by Desis Rising Up and Moving as an initial step toward transformative action. By cultivating solidarity and collective political power within our own communities, we can work towards achieving broad racial and economic equity. These recommendations, intended for both future research endeavors and community organizations, contribute to discourse on the effects of discrimination on Asian American health-seeking behaviors and healthcare utilization. They also support the development of interventions and initiatives aimed at advancing health equity and social justice.

### Electronic supplementary material

Below is the link to the electronic supplementary material.

Supplementary Material 1

## References

[1] Jeung R, Horse AY, Popovic T, Lim R, Stop (2021). AAPI hate National Report. Ethnic Stud Rev.

[2] Mitchell T, Many Black, and Asian Americans Say They Have Experienced Discrimination Amid the COVID-19 Outbreak [Internet]. Pew Research Center’s Social & Demographic Trends Project. 2020 [cited 2022 Nov 4]. Available from: https://www.pewresearch.org/social-trends/2020/07/01/many-black-and-asian-americans-say-they-have-experienced-discrimination-amid-the-covid-19-outbreak/.

[3] Takamura JC, Browne C, Jeung R, Yellow Horse AJ, Kwok D, Howard D (2022). Asian American elders: Caught in the crosshairs of a Syndemic of Racism, Misogyny, and Ageism during Coronavirus Disease 2019. Public Policy Aging Rep.

[4] Pillai D, Jeung R. The Rising Tide of Violence and Discrimination Against Asian American and Pacific Islander Women and Girls [Internet]. Stop AAPI Hate; p. 10. Available from: https://stopaapihate.org/wp-content/uploads/2021/05/Stop-AAPI-Hate_NAPAWF_Whitepaper.pdf.

[5] Timsit A. San Francisco police mark 567% increase in anti-Asian hate-crime reports in 2021. Washington Post [Internet]. 2022 Jan 26 [cited 2023 Jun 15]; Available from: https://www.washingtonpost.com/nation/2022/01/26/anti-asian-hate-crime-san-francisco-covid/.

[6] Gee GC, Spencer MS, Chen J, Takeuchi D (2007). A nationwide study of discrimination and chronic health conditions among Asian americans. Am J Public Health.

[7] Cheah CSL, Wang C, Ren H, Zong X, Cho HS, Xue X (2020). COVID-19 racism and Mental Health in Chinese American families. Pediatrics.

[8] Reny TT, Barreto MA (2022). Xenophobia in the time of pandemic: othering, anti-asian attitudes, and COVID-19. Politics. Groups and Identities.

[9] Shariff-Marco S, Breen N, Landrine H, Reeve BB, Krieger N, Gee GC (2011). MEASURING EVERYDAY RACIAL/ETHNIC DISCRIMINATION IN HEALTH SURVEYS: how best to ask the questions, in one or two stages, across Multiple Racial/Ethnic Groups?. Du Bois Rev.

[10] Ta Park VM, Dougan MM, Meyer OL, Nam B, Tzuang M, Park LG (2022). Discrimination experiences during COVID-19 among a National, Multi-lingual, Community-based sample of Asian americans and Pacific islanders: COMPASS findings. IJERPH.

[11] Nguyen TT, Criss S, Dwivedi P, Huang D, Keralis J, Hsu E (2020). Exploring U.S. shifts in anti-asian sentiment with the emergence of COVID-19. IJERPH.

[12] Xia T, Gee GC, Li J, Liu X, Dai J, Shi L et al. Associations of racial and ethnic discrimination with adverse changes in exercise and screen time during the COVID-19 pandemic in the United States. Epidemiol Health [Internet]. 2023 [cited 2023 Jun 15]; Available from: http://e-epih.org/journal/view.php?doi=10.4178/epih.e2023013. 10.4178/epih.e2023013 PMC10266926 36731474

[13] Helgadóttir B, Forsell Y, Ekblom Ö (2015). Physical activity patterns of people affected by depressive and anxiety disorders as measured by accelerometers: a cross-sectional study. PLoS ONE.

