# Supplementary material for: Asian American Women’s Experiences of Discrimination and Health Behaviors during the COVID-19 Pandemic
Source: J Immigr Minor Health. 2023 Oct 26;26(2):421–5. doi: 10.1007/s10903-023-01558-2 (PMC10937770; doi:10.1007/s10903-023-01558-2)

**SUPPLEMENTAL MATERIALS**

**Supplemental Figure 1: Frequency of specific discriminatory experiences among Asian American women overall, stratified by age (n=193)**

**Panel A: Stratified by age**


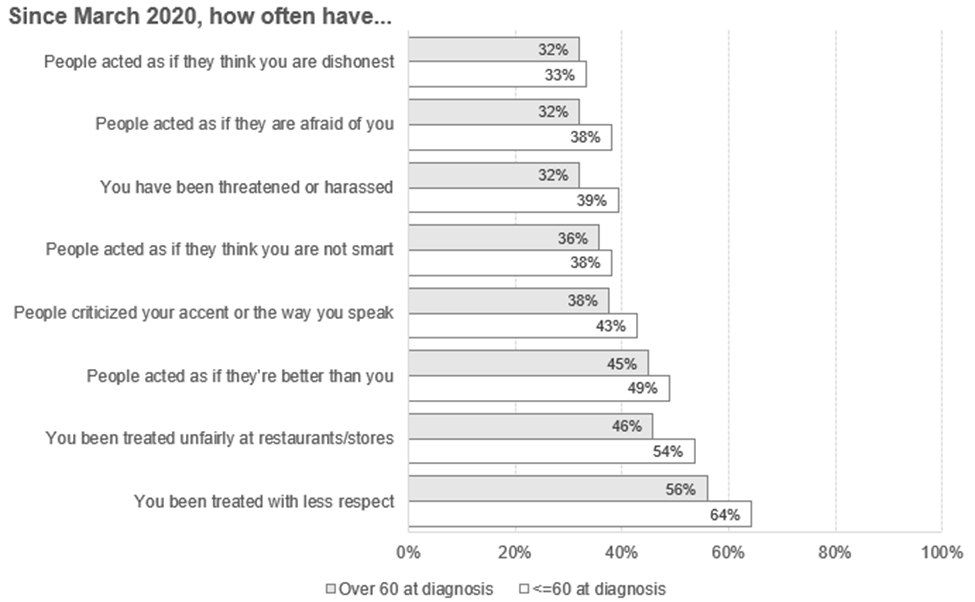

Supplement: Supplementary file 1 — Supplementary Material 1 [file 10903_2023_1558_MOESM1_ESM.docx]
